# Mechanism of Multi-Stage Degradation in Hot Bitumen of Micronized Powder Elastomeric Modifiers from Worn-Out Tire’s Rubber

**DOI:** 10.3390/polym14194112

**Published:** 2022-09-30

**Authors:** Vadim Nikol’skii, Tatiana Dudareva, Irina Krasotkina, Irina Gordeeva, Viktoriya Gorbatova, Alexandre A. Vetcher, Alexander Botin

**Affiliations:** 1 N.N. Semenov Federal Research Center of Chemical Physics, Russian Academy of Sciences, 4, Kosygin Str., 119991 Moscow, Russia; 2 Institute of Biochemical Technology and Nanotechnology (IBTN) of the Peoples’ Friendship University of Russia (RUDN), 6 Miklukho-Maklaya Str., 117198 Moscow, Russia; 3 Complimentary and Integrative Health Clinic of Dr. Shishonin, 5 Yasnogorskaya Str., 117588 Moscow, Russia; 4 N.V. Sklifosovsky Institute of Emergency Medicine, 129090 Moscow, Russia

**Keywords:** powder elastomeric modifiers, hybrid powder, high-temperature shear-induced grinding, bitumen, AFM, rheology, modification mechanism

## Abstract

For the first time, by atomic force microscopy (AFM) methods, micro- and nanofragments of micronized powder elastomeric modifier (PEM) formed at the short-term (3 min at 160 °C) interaction of PEM with hot bitumen have been demonstrated. It is the technology of high-temperature shear-induced grinding of a worn-out tire’s crumb rubber or its co-grinding with styrene–butadiene–styrene (SBS) block copolymer which provides the creation of the PEM structure inclined to rapid degradation in hot bitumen. The formation just after the preparation process of a new structure of a modified binder, more resistant to external effects, is supported by the data of rheological tests. Performance tests for a modified binder using Superpave standard adopted by the road industry for bituminous binders showed an extended temperature range, resistance to rutting, and low-temperature and fatigue cracking. The better resistance to low-temperature and fatigue cracking is certainly related to energy absorption and crack growth stopping in the presence of micron and submicron resilient PEM fragments in accordance with the mechanism of increasing impact toughness in plastics.

## 1. Introduction

Crumb rubber, obtained using the technology of cryogenic grinding or mechanical crushing at an ambient temperature of worn-out tires, is used in the road industry as a modifier of bitumen and/or asphalt mixtures [1].

One of the main aims of bitumen modification is to extend the temperature range of the bitumen and to increase the resistance to pavement defects (rutting, temperature, and fatigue cracking), to the formation of which bitumen contributes significantly [2]. When using a modifier, it is important to understand whether it can increase the bitumen resistance to permanent deformation (rutting) at high in-service pavement temperatures and whether it has an enhancing or weakening effect on the structuring and cracking processes of bitumen at intermediate and low temperatures. For example, there are AFM data showing that when bitumen cools, rigid “bee-like” structures form, which can be cracking concentrators, especially under cyclic loading [3]. Most modifiers increase the resistance of bitumen to rutting at high in-service temperatures. The improvement in pavement defect resistance at lower temperatures by modification is not so obvious [4].

When using crumb rubber (CR) as a modifier, the main features of the modification process are: the number of technological stages, apparatus design, temperature, duration of the process, and the efficiency of modification, which are mainly determined by the method of obtaining CR, its size, and morphology [5,6,7,8]. Two methods of modification are used in the application of CR. The first method is the introduction of CR directly into the mixer during asphalt mixture preparation. Most often in this case, CR replaces some fractions of the mineral components of the asphalt mixture and does not give a noticeable result in terms of improving the properties of bitumen, because the mixing time at high temperature (up to 1 min) is too short [5,8,9,10]. The second method aims to improve the rheological properties of bitumen and is a separate technological step—the preparation of rubber–bitumen binder, preceding the preparation of asphalt mixture. In this case, CR with an initial size of up to 1 mm, and in recent years up to 0.6 mm, is subjected to a sufficiently long mixing process (at least 1 hr) with bitumen at relatively high temperatures (170÷200 °C). In long-term contact with hot bitumen, some of the CR particles, mostly smaller than 200–300 μm in size, noticeably swell and break down along the cracks formed in the crushing of the worn-out tire. Nevertheless, many particles often do not undergo visible changes even after prolonged agitation. Thus, micromechanical models are developed for the complex shear modulus of rubber-modified binder, and swelled particles of the same size as the original are considered [11]. However, there are available data showing that a significant increase in the fatigue cracking resistance of modified bitumen is observed only when the amount of CR particles smaller than 75 μm is increased by at least 5% compared to the original amount. [9]. To increase the compatibility of CR with bitumen, various methods of chemical or physical–mechanical treatment are used to change the surface structure of the particles. In this case, a layer of partially regenerated rubber is formed on the surface of the particles. However, the process of bitumen modification, even in this case, requires high temperature and long mixing time [12,13,14,15].

The goal of this study was to investigate the degradation mechanism of micronized powder elastomeric modifiers (PEM) particles during interaction with hot bitumen. Micronized PEMs are produced by the high-temperature shear-induced grinding (HTSG) of CR or the co-grinding of CR and styrene–butadiene–styrene block copolymer (SBS). Such modifiers are active powders of discretely devulcanized rubber (APDDR) and hybrid (APDDR-SBS) powder. PEM is injected into the asphalt mixture during its preparation. The process of PEM production is realized in specialized equipment: a rotary dispergator. HTSG is intermediate in temperature of the grinding process (160–170 °C) among tire-rubber-recycling methods between its mechanical crushing and obtaining the regenerated rubber by devulcanization [16]. In the grinding zone of the rotary dispergator, due to high temperature and significant shear forces applied to the compressed layer of material in the modulated mode, along with a decrease in the size of the processed particles, discrete devulcanization of the rubber occurs in the locations where optimal conditions are created. It is assumed that the intermolecular bonds are disrupted and rearranged, and, in the case of co-grinding, the components are combined on the micro- and nano level. It is also noted that there is almost no change in molecular weight distribution of rubber molecules, since at such temperatures, macromolecules usually have time to relax without breaking the molecular chain. According to electron microscopy data, a PEM particle is an agglomerate of micron- and submicron-size blocks, connected by strands of different thickness (from several nanometers to several microns). According to atomic force microscopy data, the structure of PEM particles can be defined as having a strong phase heterogeneity (at the level of 0.1–0.2 μm or even less) [16,17].

Previously, the method of electron scanning microscopy (SEM) showed that after the solvent washing of binders prepared by mixing bitumen with PEM for 1–40 min in the temperature range 120–180 °C, fragments of PEM in the form of particles up to 100–200 nm or in the form of micron-sized thin films were observed [17]. However, it is important to test the presence of such fragments in the modified bitumen. In this study, comparative research by atomic force microscopy (AFM) of the surface structure of the original bitumen and PEM-modified binder prepared under temperature and time conditions close to the preparation conditions of asphalt mixtures (3 min at 160 °C) was carried out. To estimate the effectiveness of modification after a short-term interaction of PEM with hot bitumen for the original (no-aged) samples of bitumen and freshly prepared PEM-modified binders (which were not subjected to additional temperature effects), comparative performance tests in a wide temperature range by using Superpave standards adopted by the road industry for bitumen binder were conducted [13,18,19].

## 2. Materials and Methods

### 2.1. Materials

The following materials were investigated in this work:

- Micronized powder elastomeric modifiers (PEM): APDDR and hybrid (APDDR-SBS) powder, obtained by high-temperature shear-induced grinding of crumb rubber in a rotary dispergator. APDDR was produced by grinding of CR of worn-out tires and hybrid powder by co-grinding of CR and SBS L 30-01 (content of bound styrene, wt%—29-31; molecular weight—around 78 KD). The APDDR and hybrid powder were homogeneous black powders. The properties of PEM are listed in Table 1. The specific surface was determined by the BET method at T = 77 K using the adsorption analyzer of surface area “NOVA 1200e” (Quantachrome Instruments, Ltd.; Boynton Beach, FL, USA). Particle size distribution parameters were determined using wet laser diffraction using “ANALYSETTE 22 NanoTec plus” (Fritsch GmbH – Mahlen und Messen, Idar-Oberstein, Germany).

- Blown bitumen grade BND 60/90 was used as a basis for preparation of modified binders for AFM research and performance testing.

- Blown bitumen grade BND 100/130 was used for preparation of modified binders and performance testing.

The coding and properties of bitumen samples are shown in Table 2.

Modified binders were prepared:

- For AFM research by mixing, 10÷20 wt.% APDDR or hybrid 80/20 powder (by co-grinding of 80 wt.% CR and 20 wt.% SBS L 30-01) with 90÷80 wt.% bitumen Bit-A heated to 160 °C with 180 rpm of paddle stirrer (IKA HB10 DIGITAL) for 1 min;

- For performance testing by mixing, APDDR or hybrid 80/20 or hybrid 95/5 powder (cogrinding of 95 wt.% CR and 5 wt.% SBS L 30-01) with bitumen heated to 160 °C with 600 rpm of paddle stirrer for 3 min. The coding and composition of modified binder samples are presented in Table 3.

### 2.2. Research Methods

#### 2.2.1. AFM Study

Research of structural and morphological features of the bitumen and MB sample’s surface was carried out on an NtegraPrima AFM (NT-MDT Spectrum Instruments, LLC; Moscow, Russia) in a semicontact mode. AFM topography and phase imaging were obtained at room temperature at a scanning speed of 0.6÷1 Hz. Gold-plated silicon cantilevers were used (rounding radius 10 nm; resonance frequency 150÷240 kHz).

#### 2.2.2. Rheological Study

Rheological tests were carried out on a dynamic shear rheometer MCR 702e (Anton Paar, GmbH, Gratz, Austria) using a parallel geometry measuring system:

- 25 mm for a multiple stress creep recovery (MSCR) test [20] (10 cycles, creep phase 1 s, recovery phase 9 s at different stress levels—0.1, 3.2, 6.5, 10, and 12 kPa at 64 °C) and for a dynamic (oscillatory) shear test at an angular frequency of 10 rad/s and shear strain 12% according to [21];

- 8 mm diameter for a linear amplitude sweep (LAS) test [22] (at 0.1 Hz in oscillatory mode in increasing strain: 0.1%, 1% and further up to 30% in steps of 1% (total 3100 cycles) at 16 °C).

The measuring gap during the tests was 1 mm for the MSCR test and for the dynamic shear test and 2 mm for the LAS test. The specimens were poured into silicone molds of 25 or 8 mm diameter. Rheological tests of the samples were carried out not earlier than 15 min and not later than 2 h after placing them into the molds. The samples were deposited in a rheometer at 58 °C.

Low-temperature-cracking parameters were determined on an Asphalt Binder Cracking Device (ABCD) (Infratest, LLC; Moscow, Russia) under static conditions according to the ABCD test [23] while cooling four parallel samples in a climatic chamber at a rate of 18÷20 °C/h.

## 3. Results and Discussion

### 3.1. AFM Study

Atomic force microscopy has been used for more than 25 years to study the microstructure of bitumen. Particular attention of the researchers was focused on the so-called “bee-like” structure [24]. Creep measurements demonstrated that the microstructure of the “bee-like” phase has 40–50% higher stiffness than the surrounding matrix phase [25]. Studies of the binders’ resistance to low-temperature cracking after applied load showed the appearance of cracks at the “bee”-matrix phase interface [3], which allowed the visualization of theoretical ideas justifying the possibility of binder cracking under climatic factors and transport loading due to its inherent heterogeneity [26].

**Figure 1:**
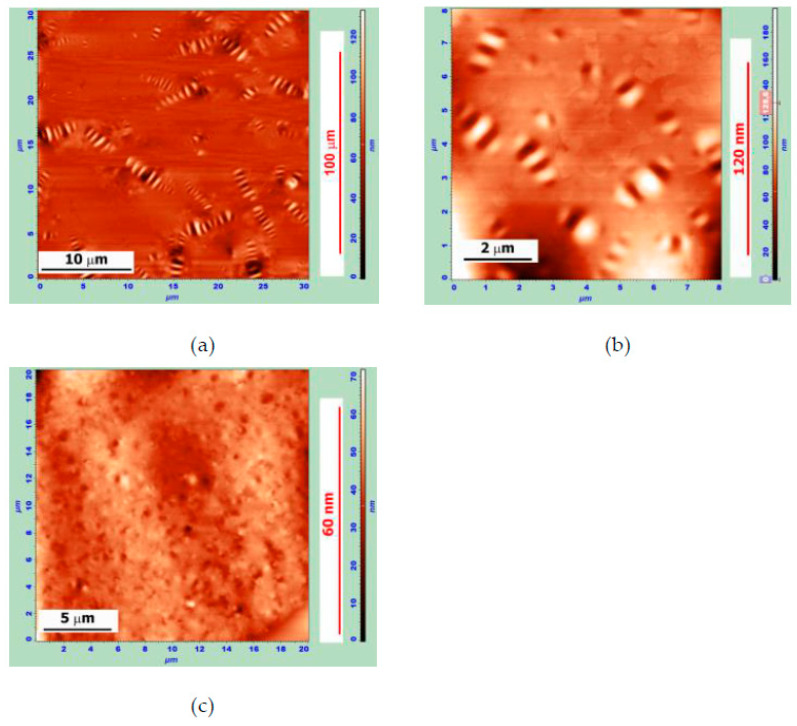
AFM images of the surface topography of (**a**) the sample of Bit-A, obtained 2 days after sample preparation (30 × 30 μm, Z-coordinate—130 nm); (**b**) the sample of MB of 90 wt.% Bit-A+ 10 wt.% APDDR, obtained 2 days after sample preparation (8 × 8 μm, Z-coordinate—190 nm); (**c**) the sample of MB of 80 wt.% Bit-A+ 20 wt.% APDDR obtained 1.5 months after sample preparation (20 × 20 μm, Z-coordinate—70 nm).

Figure 1a shows the surface topography of bitumen Bit-A, which illustrates the structural heterogeneity of bitumen and the presence of a pronounced microheterogeneity in it. The surface of the bitumen sample examined 2 days after preparation showed the presence of a “periphase” surrounded by “bee-like” structures with a maximum size of 5–7 μm, with a tendency to form “star bee-like” structures. Storage of this bitumen at ambient temperature for 1.5 months led to an increase in the maximum size of the “bee-like” structures by 1.5–2 times—up to 15–20 μm.

The ©ntroduction of APDDR into bitumen primarily had an effect on the formation of a “bee-like” structure. 

For MB with 10 wt.% APDDR content, a significant decrease in the length of “bees” to the maximum 2–3 μm was observed (Figure 1b). When the APDDR content in the MB was increased to 15 wt.% and higher, no “bees” were observed even after 1.5 months of sample storage (Figure 1c).

The structure of the fragmented PEM particles observed using AFM was compared with the SEM data for the modified binder, where the APDDR content was 15 wt.%. The measurements were carried out 2 days after MB preparation (3 minutes’ mixing at 160 °C).

**Figure 2:**
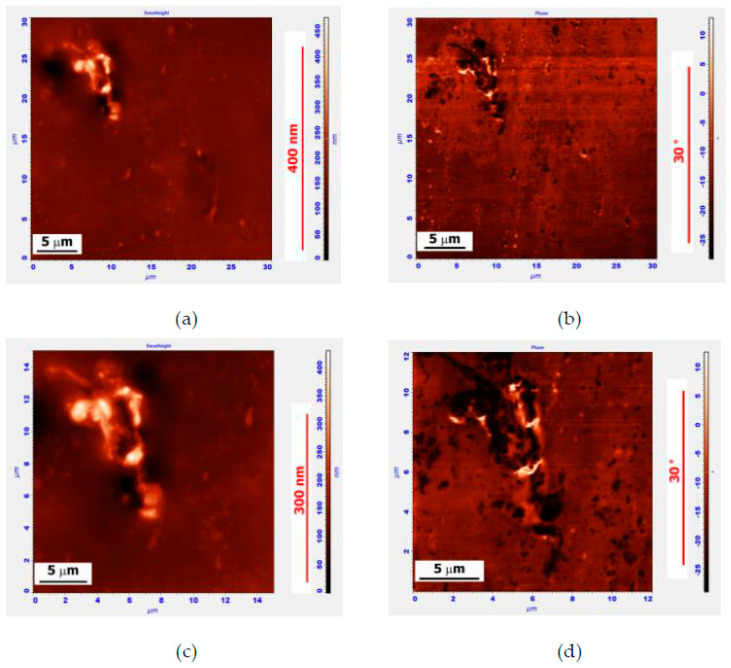
AFM surface images of the MB sample of 85 wt.% Bit-A + 15 wt.% APDDR, obtained 2 days after sample preparation. Topography (left), phase image (right): (**a**,**b**)—30 × 30 μm, (**a**) Z-coordinate—470 nm; (**c**,**d**)—12 × 12 μm, (**c**) Z-coordinate—470 nm.

In Figure 2a,b, one can see an elongated APDDR particle of the size of about 10 μm. When the image is zoomed in (Figure 2c,d), it is clearly seen that the particle consists of two different-sized fragments: the upper one is about 4÷5 µm long, and the lower one is about 2 µm. These fragments are connected by strands up to 2µm-long and a few tenths of a micron thick. We can also assume that the upper fragment of this APDDR particle consists of at least three parts. It can be hypothesized that this particle is the decay product of a larger particle, whose fragments were interconnected by strands.

**Figure 3:**
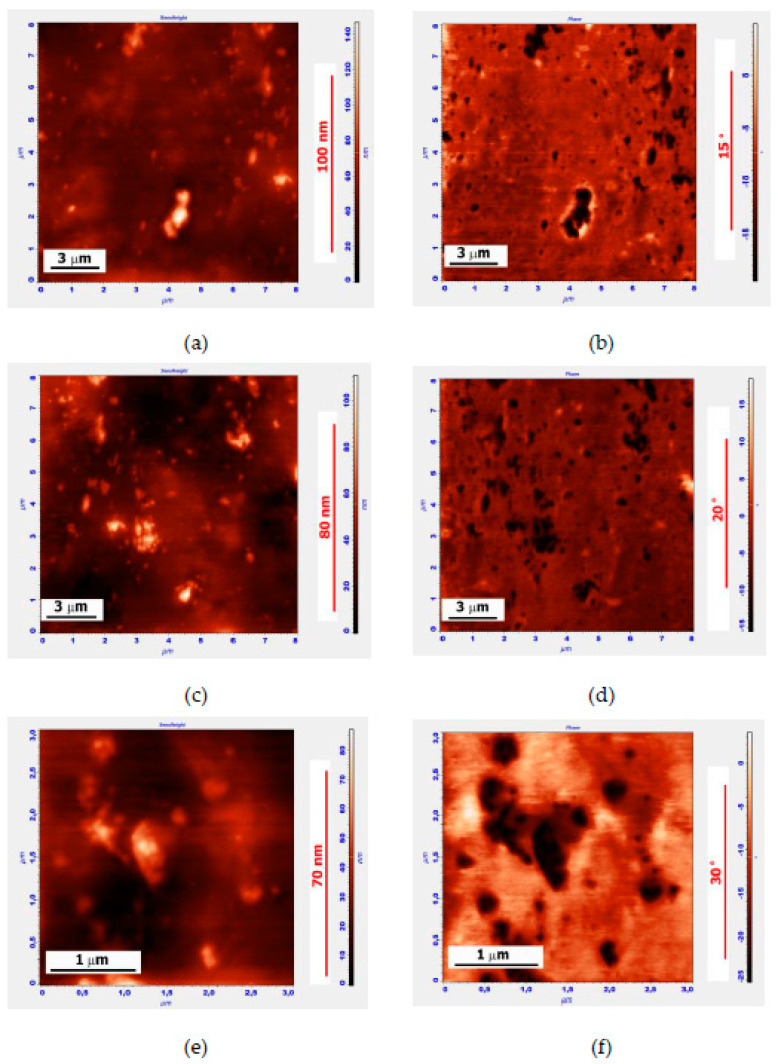
AFM surface images of the MB sample of 85 wt.% Bit-A + 15 wt.% APDDR, obtained 2 days after sample preparation. Topography (left), phase image (right): (**a**,**b**)—8 × 8 μm, (**a**) Z-coordinate—140 nm; (**c**,**d**)—8 × 8 μm, (**c**) Z-coordinate—110 nm; (**e**,**f**)—3 × 3 μm, © Z-coordinate—90 nm.

Smaller fragments of the original APDDR particles of 100÷1000 nm are clearly visible in Figure 3. Similar particles of the same size and similar (self-similar) agglomerative structure were observed in SEM images after washing the MB with solvent [17].

**Figure 4:**
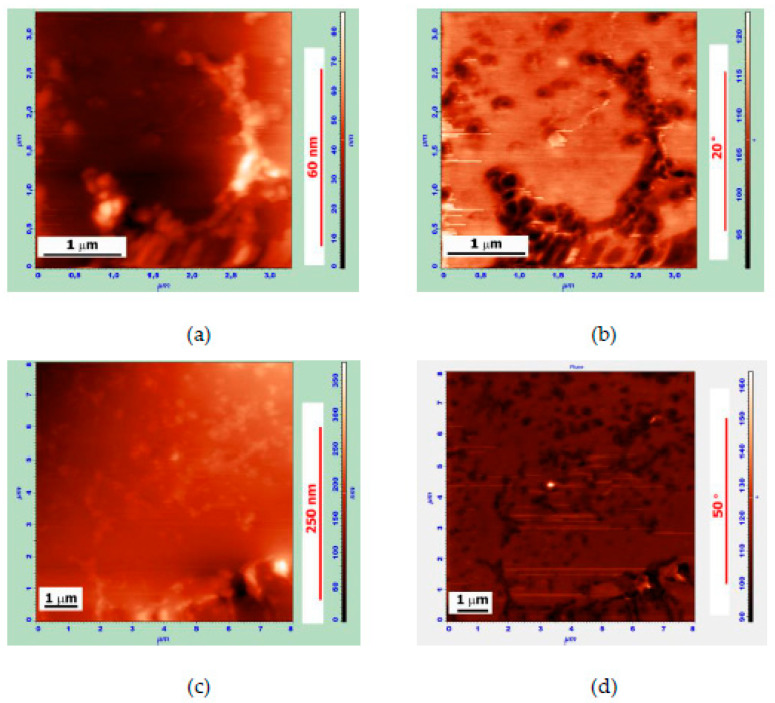
AFM images of the MB surface with the gel structure area. Topography (left), phase image (right): (**a**,**b**)—3 × 3 μm, (**a**) Z-coordinate—85 nm; (**c**,**d**)—8 × 8 μm, (**c**) Z-coordinate—375 nm.

The images in Figure 4 illustrate the stage preceding the separation of the 50÷200 nm APDDR fragments from a larger particle (Figure 4a,b) and the formation of the spatial structure (Figure 4c,d). The data presented in Figure 3 and Figure 4 indicate the formation of a physical spatial network of rubber fragments. Since the distances between the fragments are comparable to their fragment sizes, the percolation threshold’s conditions are satisfied.

**Figure 5:**
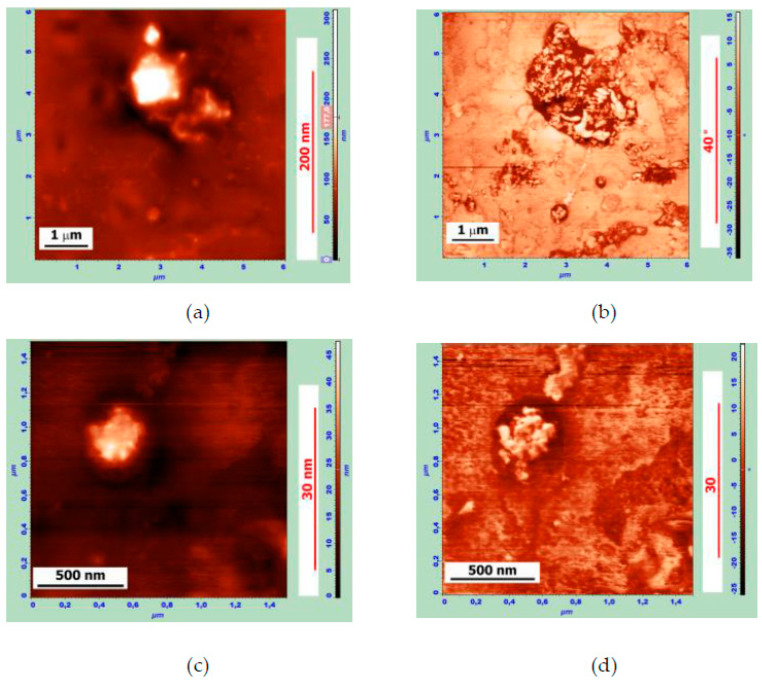
AFM images of the surface of the sample of the MB with composition of 85 wt.% Bit-A +15 wt.% hybrid powder, obtained 2 days after sample preparation. Topography (left), phase image (right): (**a**,**b**)—6 × 6 μm, (**a**) Z-coordinate—310 nm; (**c**,**d**)—1.4 × 1.4 μm, (**c**) Z-coordinate—50 nm.

Figure 5 shows AFM images of the MB surface (the MB is based on Bit-A and hybrid powder; the Bit-A/hybrid powder is 85/15 wt.%). The measurements were carried out two days after sample preparation.

In a series of zoomed images, the different stages and mechanisms of hybrid particle disintegration can be observed. In Figure 5a,b at the top left, a round-shaped hybrid particle of 2–3 μm in size can be observed connected by strands with smaller fragments up to 0.2 μm in size. One such fragment is shown in Figure 5c,d. The appearance of these fragments correlates very well with SEM images of the APDDR fragments [17]. Elements of an agglomerative structure are also traced.

**Figure 6:**
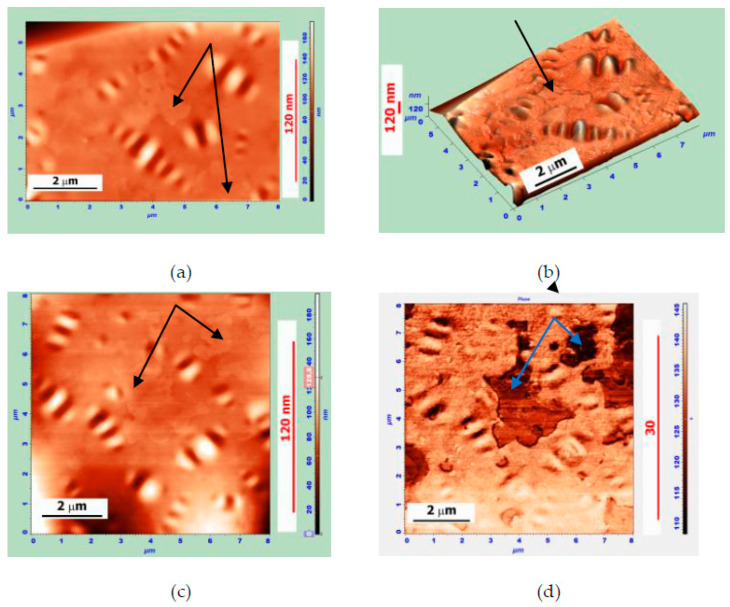
AFM surface images of the MB sample of composition 90 wt.% Bit-A + 10 wt.% APDDR, obtained after 2 days after sample preparation. (**a**,**c**)—topography, (**b**)—AFM-3D image; (**d**)—phase image. (**a**,**b**)—8 × 6 μm, (**a**) Z-coordinate—175 nm; (**c**,**d**)—8 × 8 μm, (**c**) Z-coordinate—195 nm. Thin films are indicated by arrows.

The AFM images of thin films observed on the surface of the MB with a 90 wt.% Bit-A+10 wt.% APDDR composition are shown in Figure 6. In our opinion, the separation of such films occurs as a result of multidirectional swelling forces from the surface of microblocks with a denser structure than others. In our opinion, these images have similarities with the fragments of APDDR particles in the form of films observed earlier using electron scanning microscopy [17]. Figure 6b shows a three-dimensional image of the MB surface, from which we can see that the film is partially located on the bee structure. Thus, it is verified that the films were formed by the rapid decay of PEM particles, because the formation of the “bee” takes longer.

The results of AFM studies confirm the main conclusions drawn based on SEM studies [17]: already at the early stage of interaction, PEM particles break down into micro- and nanofragments with an agglomerative structure, similar (self-similar) to that of the original modifier particles given in [17], as well as present in the form of thin films. Additional information obtained on the basis of AFM images concerns the formation of gel structures on the basis of broken PEM particles. Additional information obtained on the basis of AFM images concerns the formation of gel structures on the basis of the broken PEM particles. Such a spatial network of nano- and microfragments should hinder the processes of diffusion and crystallization of the waxes present in bitumen. The result is the disappearance or a significant decrease in the size of “bee-like” formations, which is observed at PEM concentrations above 10 wt.%. A more homogeneous structure of the modified binder is created. 

### 3.2. Rheological Tests

The structure of binders resulting from the interaction of rubber particles (APDDR) and hybrid particles (APDDR-SBS) obtained by high-temperature shear grinding with hot bitumen determines the rheological and, therefore, the performance properties of such binders. Rheological tests according to Superpave standards were performed on aged binder specimens. The aging was carried out for 85 min at 163 °C in a rolling thin-film oven (RTFO) [27] and 20 h at 100 °C in a pressure-aging vessel (PAV) [28]. This aging simulates the processes of asphalt concrete mixture preparation and as well as the 7-year operation service of the pavement. Earlier, it was shown that aged samples of PEM-modified bitumen at a PEM concentration of 12–15 wt.% in the binder showed an improvement in bitumen resistance to all types of pavement defects in the whole range of operating temperatures. However, it was noted that reducing the PEM concentration to 10 wt.% and less led to some improvement in low- and high-temperature parameters but worsened some parameters of fatigue-cracking resistance of the modified samples compared to bitumen [29]. These data justified the PEM concentration in bitumen for rheological tests as 12.5 wt.%. In this case, rheological tests were conducted for unaged bitumen to see the change in binder properties immediately after the introduction of the modifier.

Among the parameters of resistance to rutting the most promising for the characteristic of bituminous binders is considered parameter J_nr_ (unrecoverable creep compliance), determined in the test for resilience to multiple cycles of creep–recovery [20]. The parameter J_nr_ is calculated as the ratio of the average unrecovered creep strain over 10 test cycles to the applied stress level. The second important parameter is the elastic recovery in percent at a given load (R_3.2)_ calculated as the average value of the elastic recovery for 10 cycles of creep–recovery at a 3.2 kPa stress level.

Table 4 shows the data of unrecoverable creep compliance (J_nr_) and elastic recovery at a 3.2 kPa stress level (R_3.2_) of the MSCR test for two bitumen and modified binders, the composition of which is given in Table 3. Additionally, in Table 4 are the data for the upper operating temperature of the no-aged bitumen binders, which is defined from a dynamic (oscillatory) shear test [21] as the temperature at which rutting parameter G*/sinδ equals 1 kPa.

A test for multiple stress creep recovery was conducted to characterize the samples in terms of resistance to rutting during the operation of the pavement in the summer under the influence of moving traffic. The introduction into both bitumen samples of a 12.5% powder elastic modifier (PEM) led to a sharp decrease in unrecoverable creep compliance (J_nr_) at almost all load levels. Increasing the SBS content of PEM reduced J_nr_ and increased elastic recovery (R) (Table 4 shows the elastic recovery (R_3.2_) for the 3.2 kPa stress level) both compared to bitumen and to SBS-free APDDR. MSCR tests carried out early (see [29], for example) for RTFO-aged bitumen samples at 64 °C showed that the elastic response was no more than 5–6%, whereas for modified binders, the elastic response exceeded 70%, which showed that the final formation of the spatial mesh occurs during the time the modified asphalt mixture is brought to the paving site. J_nr_ values after RTFO aging ensure that the modified binder can be used for pavements with maximum traffic. The good resistance to multiple cycles of creep and recovery at a sufficiently high test temperature (64 °C) of Bit-B, which has a much higher penetration compared to Bit-A, seems to be due to differences in production technology and chemical composition.

The resistance to low-temperature cracking was determined by the ABCD method. The results are presented in Table 5.

From Table 5, it can be seen that the specimen-cracking temperature (T_ABCD_) decreased with the introduction of PEM. The greatest decrease (9.8 °C) was observed for the hybrid-modified binder with 20 wt.% SBS. Only a slight decrease in fracture temperature (up to 0.5 °C) was observed for the hybrid powder with 5 wt.% SBS compared to the SBS-free APDDR-modified binder, which decreased the T_ABCD_ of both bitumens by about 4 °C. It has previously been shown that the decrease in the fracture temperature in the ABCD test using PEM compared to bitumen is also characteristic of aged samples [29]. At the same time, the ABCD data show that at the moment of fracture, the modified samples have a much higher fracture stress (σ) than the original bitumen. Thus, it is confirmed that a sufficiently short mixing time (in this case, 3 min at 160 °C) already leads to the formation of a new binder structure.

Data of Table 4 and Table 5 show a significant expansion of the operating temperature range, as well as an increase in resistance to rutting and low-temperature cracking.

**Figure 7:**
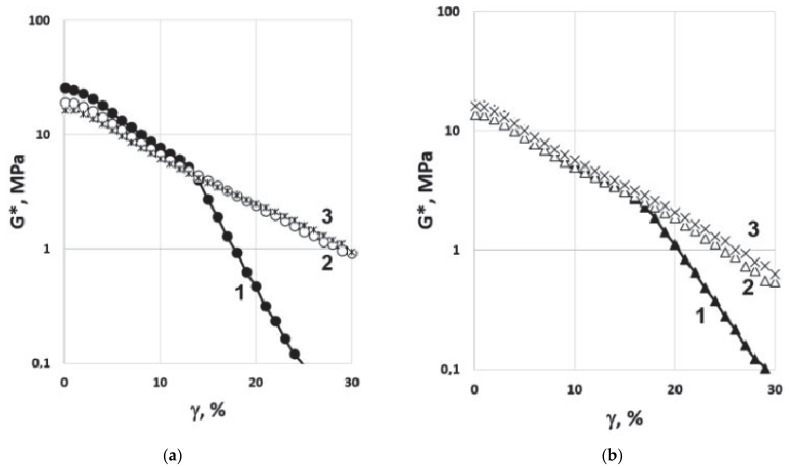
Plots of the complex modulus (G*) versus shear strain (γ) in the LAS test at temperature 16 °C: (**a**) Bit-A—1; MB-A(0)—2; MB-A(20)—3; (**b**) Bit-B—1; MB-B(0)—2; MB-B(5).

The resistance of specimens to fatigue cracking was evaluated using a linear amplitude sweep (LAS) test. Figure 7 shows the dependences of complex modulus (G*) versus shear strain (γ) recorded during testing at 16 °C. It can be seen that for the modified binders, there was no sharp drop in the complex modulus (G*), which was observed for both bitumen samples at γ of 12–18%. This may indicate the formation of a spatial network in the modified binders, the existence of which is most pronounced during fatigue tests.

**Figure 8:**
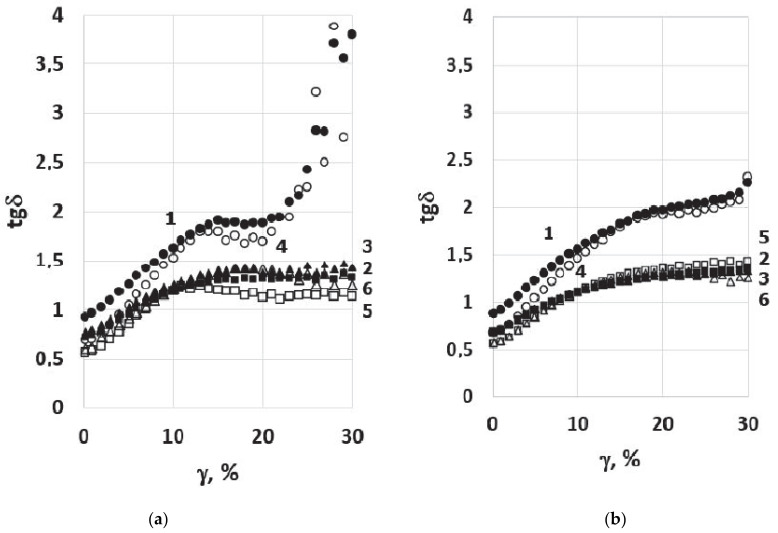
Plots of the tgδ versus shear strain (γ) in the LAS test at temperature 16 (1, 2, and 3) and 7 °C (4, 5, and 6) for Bit-A (**a**—1 and 4); MB-A(0) (**a**—2 and 5); MB-A(20) (**a**—3 and 6); Bit-B (**b**—1 and 4); MB-B(0) (**b**—2 and 5); MB-B(5) (**b**—3 and 6).

Figure 8 shows the dependence tangent of phase angle (tg¦Ä) versus shear strain (γ) recorded during testing at 16 and 7 °C. As can be seen, the modified binders in all cases show less sensitivity to cyclic deformation.

Table 6 shows the number of cycles to failure (N_f_) at strains of 2.5 and 5%, calculated according to [22] based on LAS test data. As can be seen, the modification significantly improved the resistance to cyclic strain. Recall that Table 6 shows the data obtained for no-aged specimens of the modified binders. After RTFO and after PAV aging, the best results were observed for the modified binder based on hybrid PEM particles. 

The comparative rheological tests of bitumen samples and PEM-modified binder produced in conditions close to the temperature–time conditions of road mixture production (3 min mixing at T = 160 °C) and not subjected to additional temperature influence showed that even such a short time of interaction of the hot bitumen and PEM provides an expansion of the performance temperature range of modified bitumen and its increased resistance to cyclic loads. 

The improvement in rheological indicators confirms that the rapid degradation of micronized powders of elastic modifiers into micro- and nanofragments leads simultaneously to the formation of a new structure of the modified binder that is more resistant to external influences. It can also be assumed that the improvement in low-temperature and fatigue-cracking resistance may be due to energy absorption and crack growth stopping in the presence of micron and submicron PEM elastic fragments in accordance with the mechanism of increasing impact toughness in plastics [30,31,32].

The results obtained confirm the effective recycling of worn-out tire rubber by the HTSG method in obtaining PEM for further use to modify bitumen directly in the production of road asphalt mixtures or to reduce the time and energy costs for the preparation of modified bitumen binder for road construction.

## 4. Conclusions

In this paper, the structure and performance properties of bitumen and modified binders obtained as a result of the short-term interaction of hot bitumen and micronized powder elastomeric modifiers (PEM) were investigated. PEM was produced by the high-temperature shear-induced grinding of a worn-out tire’s crumb rubber or its co-grinding with butadiene styrene thermoplastic elastomer. 

Atomic force microscopy (AFM) studies have shown that a short-term interaction between hot bitumen with PEM particles having a highly developed surface leads to their degradation into numerous micro- and nanofragments. It is hypothesized that the rapid degradation of PEM particles occurs under the action of multidirectional swelling forces in locations of stressed-bond concentration or locations of rubber discrete devulcanization. An agglomerative structure of fragments similar (self-similar) to the structure of the original particles of the modifier (given in [17]) forms. The fragments in the form of thin films apparently form when the swollen rubber peels off the surface of the particle.

AFM studies also showed the formation of a more homogeneous binder structure at PEM concentrations greater than 10 wt.% by significantly reducing the length of the “bees” up to the disappearance of “bee-like” units.

The results of rheological tests of bitumen and freshly prepared (not subjected to additional temperature treatment) samples of modified binder showed an improvement in binder performance at a wide range of temperatures and load levels, including: 

- Expansion of the operating temperature range;

- Increased resistance to permanent deformation (rutting) at high temperature, including high creep resistance, sensitivity to load increase, and elastic recovery;

- Improved resistance to low-temperature and fatigue cracking.

The improvement in rheological performance confirms that the rapid degradation of micronized powders of elastic modifiers into micro- and nanofragments leads simultaneously to the formation of a new binder structure that is more resistant to external influences. It can also be assumed that the improvement in cracking resistance in modified binders is due to energy absorption and crack growth being stopped by the spatial network of micron and submicron resilient PEM fragments.

The results obtained clarify the effectiveness of PEM in the most cost-effective way: the injection of PEM into the asphalt mixture just at the time of its preparation.

Further work will be aimed at studying the structure and properties of mastics containing PEM and assessing the PEM effect on the interfacial interaction between modified binder and mineral filler.

## Figures and Tables

**Table 1:** The PEM properties.

PEM	Particle Size Distribution Parameters, μm			Specific Surface, m^2^/g
	D_10_	D_50_	D_90_	
APDDR	40	140	300	0.49
Hybrid (APDDR+SBS)	50	160	340	0.45

**Table 2:** Properties of bitumen.

Penetration Grade	BND 60/90	BND 100/130
Bitumen code	Bit-A	Bit-B
Penetration@ 25 °C, dmm	60	110
Softening Point, R&B, °C	48	44
Fraass Breaking Point, °C	−18	−25

**Table 3:** The coding and composition of modified binder samples.

Modified Binder Code	Modified Binder Composition,%				
	Bit-A	Bit-B	APDDR	Hybrid80/20	Hybrid95/5
MB-A(0)	87.5		12.5		
MB-A(20)	87.5			12.5	
MB-B(0)		87.5	12.5		
MB-B(5)		87.5			12.5

**Table 4:** The data on unrecoverable creep compliance (J_nr_) under different stress levels and elastic recovery at 3.2 kPa stress level (R_3.2_) (MSCR@64 °C) and upper operating temperature of no-aged bitumen binders (**T@** G*/sinδ = 1 kPa).

Binder		Bit-A	МВ-A(0)	МВ-A(20)	Bit-B	МВ-B(0)	МВ-B(5)
		J_nr_, kPa^−1^					
**Stress level τ, kPa**	0.1	6.4	0.9	0.3	4.4	0.5	0.3
	3.2	8.2	1.4	0.5	7.6	0.9	0.6
	6.5	9.2	1.7	0.7	9.4	1.2	0.8
	10	11.4	1.9	0.8	11.5	1.4	0.9
	12	47.3	2.1	0.9	13.9	1.5	1.1
**Elastic recovery R_3.2_,%**		0	11	23	0	18	27
**T@** G*/sinδ = 1 kPa		64.7	85.2	91.3	67.3	84.7	89.2

**Table 5:** ABCD test results.

Binder	Bit-A	MB-A(0)	MB-A(20)	Bit-B	MB-B(0)	MB-B(5)
**T_ABCD_,** **°** **C**	−33.2	−37.1	−43	−38	−42.2	−42.7
**σ, MPa**	1.6	2.8	4.0	1.8	2.6	2.7

**Table 6:** LAS test results at temperatures 16 and 7 °C: number of cycles to failure (N_f_) (VECD analysis) at 2.5 and 5% strain.

Binder		Bit-A	MB-A(0)	MB-A(20)	Bit-B	MB-B(0)	MB-B(5)
**N_f2,5%_**	16 °C	54,000	353,500	257,500	68,600	3,480,000	1,840,000
	7 °C	7350	47,500	28,700	7830	189,000	63,400
**N_f5%_**	16 °C	4500	17,000	10,600	4600	110,900	56,500
	7 °C	325	1200	780	290	3700	1300

## Data Availability

The data presented in this study are available on request from the corresponding author(s).
